# Pediatric dentinogenic ghost cell tumor: a case report and review of the literature

**DOI:** 10.3389/fonc.2025.1665311

**Published:** 2025-11-25

**Authors:** Linlin Wang, Jingchao Han, Jianli Xie, HongSheng Tian

**Affiliations:** 1 Medical imaging department, Jinan Stomatological Hospital, Jinan, Shandong, China; 2 Department of Prosthodontics, Jinan Stomatological Hospital, Jinan, Shandong, China; 3 Medical imaging department, Affiliated Hospital of Shandong University of Traditional Chinese Medicine, Jinan, Shandong, China

**Keywords:** pediatric, ghost cell, dentinogenic ghost cell tumor, CBCT, COC

## Abstract

Dentinogenic ghost cell tumor (DGCT) is a rare odontogenic neoplasm that presents with nonspecific clinical manifestations and imaging features. It is more prevalent in elderly patients, with cases in children being extremely rare. This article presents a case study of a 10-year-old male patient who exhibited symptoms of swelling and pain in the left cheek for a duration of two weeks. Cone beam computed tomography (CBCT) demonstrated a hypodense lesion involving the left maxilla, with extension into the maxillary sinus and buccal cortical expansion. The patient underwent decompression, and histopathological examination of the intraoperative specimen suggested a diagnosis of calcifying odontogenic cyst (COC). One year after decompression, the patient underwent a tumor resection and the diagnosis of DGCT was confirmed by the post-operative pathology. Six months after tumor resection, CBCT showed complete bone remodeling in the lesion area. The patient is currently undergoing regular follow-up. This case provides an important reference for the diagnosis and treatment of pediatric DGCT, helping clinicians to develop individualised treatment plans.

## Introduction

Dentinogenic ghost cell tumor (DGCT) is a benign tumor of local aggressiveness characterised by the presence of ameloblastoma-like epithelium in the interstitial tissue of mature connective tissue, shadow cells formed by abnormal keratinisation, and variable amounts of abnormally proliferated dentin-like material (1). The age of onset for DGCT ranges from 8 to 80 years, with a higher proportion in older people, and it is very rare in children (2). This article reports a case of DGCT in a 10-year-old child and provides an analysis of the clinical manifestations, imaging features and treatment methods in conjunction with the relevant literature.

## Case description

This study was approved by the Medical Ethics Committee of Jinan Stomatological Hospital (JNSKQYY-2022-026 November 15, 2022).

A 10-year-old boy presented with a swollen and painful left cheek for 2 weeks. Oral and intramuscular antibiotics were ineffective. He was admitted to the hospital with a diagnosis of “left maxillary mass” based on outpatient evaluation. Physical examination revealed asymmetry in the patient’s jaw and face, with swelling and tenderness in the left cheek area. The skin color was normal, there was no cyanosis of the lips, and no significant tenderness was noted anterior to both ear screens. Mouth opening range and pattern were normal. 23 had not erupted, 63 was retained without mobility. There was significant swelling in the left maxillary vestibule, slight redness and swelling of the gums, and tenderness and fluctuation could be felt. Percussion tests and cold tests for 22, 24, 25, 26, and 27 were normal. No redness or swelling was observed at the openings of the parotid ducts bilaterally; clear fluid was secreted upon compression, and no enlarged lymph nodes were palpable in the maxillofacial and neck region. Laboratory tests showed no abnormalities.

**Figure 1:**
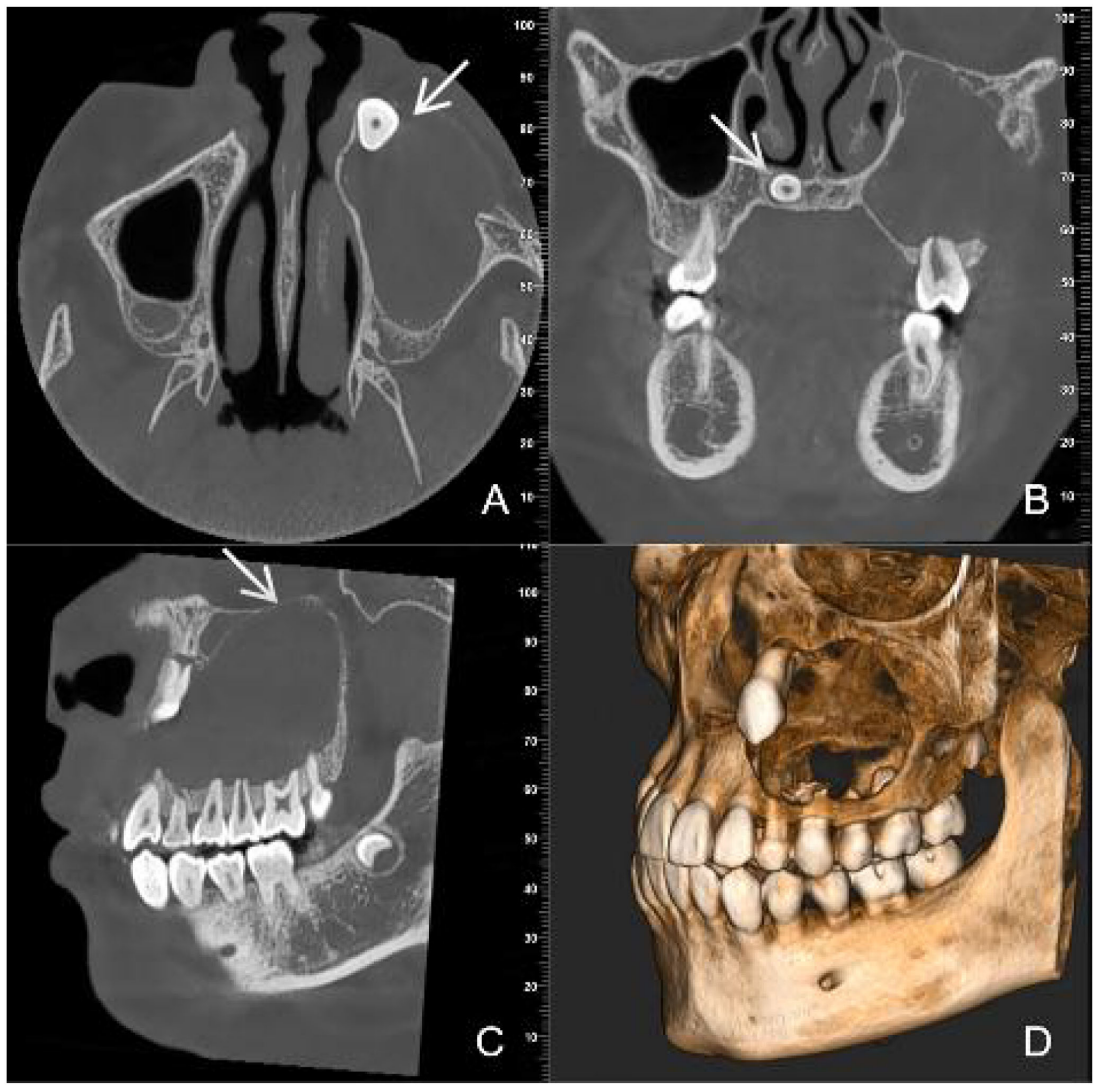
Preoperative CBCT images **(A)** (axial view) **(B)** (coronal view) **(C)** (sagittal view) **(D)** (three-dimensional reconstruction) show a large, low- density cyst with clear boundaries [images **(C)** white arrow]. An impacted tooth 23 [images **(A)** white arrow] with large irregular high-density masses is present within the lesion. A supernumerary tooth [images **(B)** white arrow] is observed in the maxillary palate slightly to the right of the midline.

**Figure 2:**
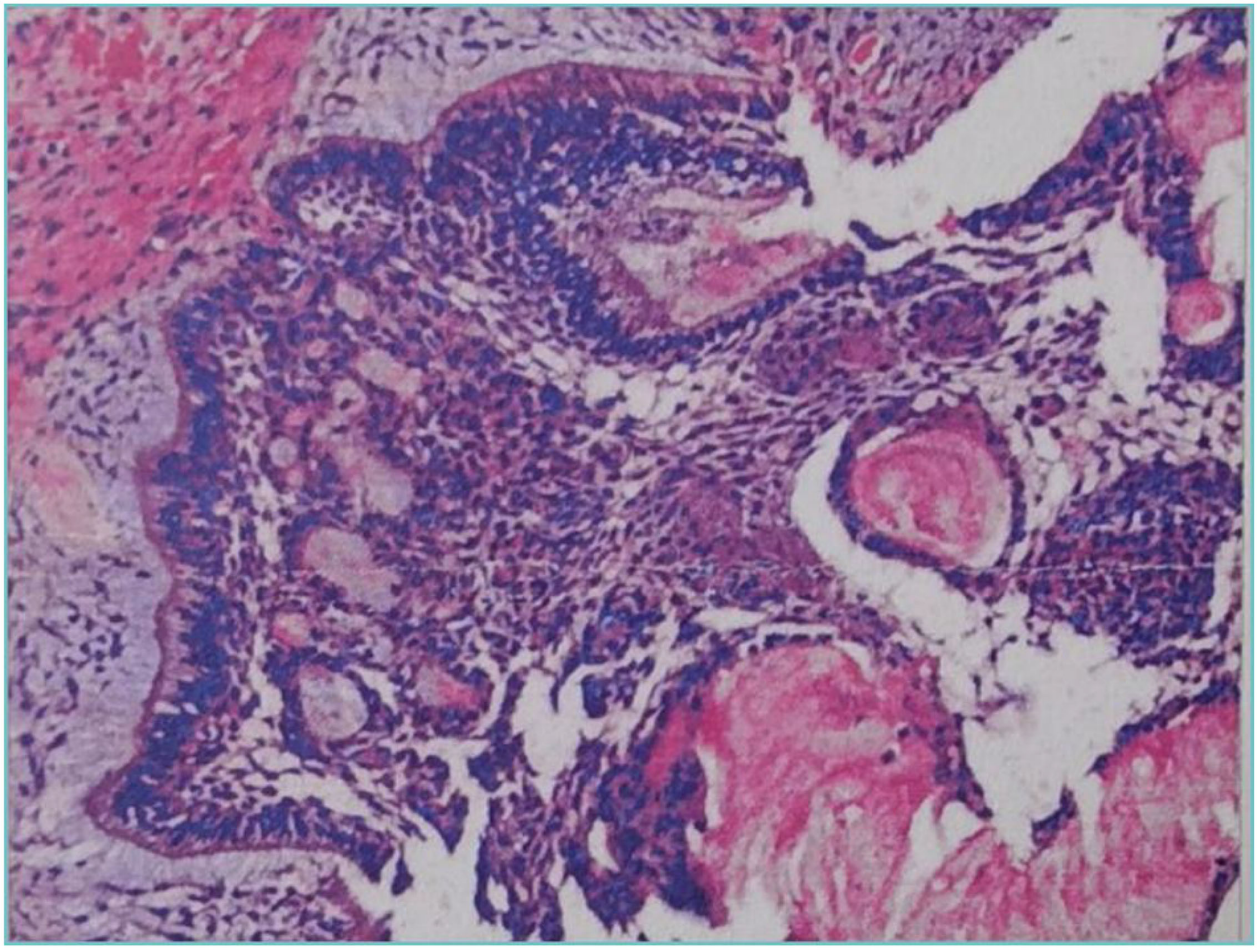
Postoperative pathology of decompressive surgery show tissue containing ameloblastoma-like epithelium and shadow cells.

CBCT images showed a hypodense lesion within the left maxillary bone extending into the maxillary sinus and buccal side. The size was approximately 40mm×30mm×23mm with clear borders. The buccal bone wall was discontinuous and there were large irregular high-density masses within the cyst. The left maxillary sinus was elevated. Tooth 63 was retained and tooth 23 was impacted in the anterior wall of the maxillary sinus. Partial external root resorption of teeth 24. 25. 26. 27 were observed. A supernumerary tooth was seen in the maxillary palate slightly to the right of the midline (**Figure 1**). Considering the young age of the patient and the large extent of the lesion, a “decompression surgery + extraction of retained primary tooth 63 + extraction of the supernumerary tooth” was performed. Intraoperative observation revealed that the mass was cystic, with thick cyst paries and brown fluid that had already drained. Part of the cyst paries tissue was sent for pathological examination. Inside the cyst cavity, irregular necrotic bone-like tissue was completely removed and sent for pathological examination. Pathological examination: Cyst paries-like tissue lined with epithelium containing ameloblastoma-like epithelium and shadow cells, the latter is calcification, with dentin tissue. Pathological diagnosis: Calcifying odontogenic cyst of the left maxillary bone (**Figure 2**).

**Figure 3:**
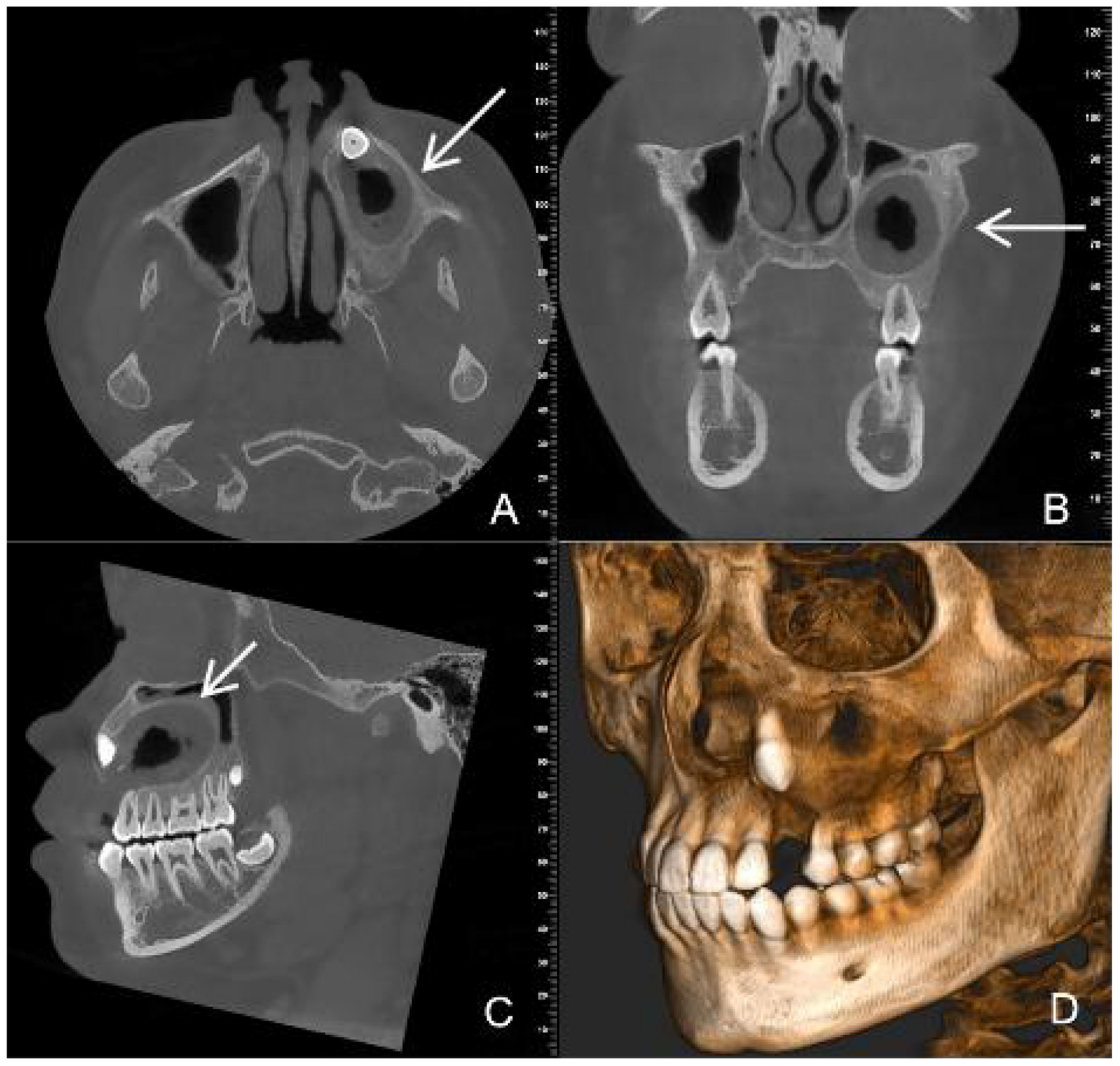
CBCT images six months after decompression surgery **(A)** (axial section) **(B)** (coronal position) **(C)** (sagittal position) **(D)** (three-dimensional reconstruction) show that the lesion size has become smaller and the bone repair area (white arrow) more obvious.

After post-operative irrigation and dressing change, wearing of a plug, regular check-ups. CBCT images at six months after decompression surgery showed that the lesion size was approximately 32mm×23mm×20mm, with clear boundaries, slightly thickened surrounding bone walls, root development 23 and periapical bone repair of teeth 24, 25, 26, 27 (**Figure 3**).

**Figure 4:**
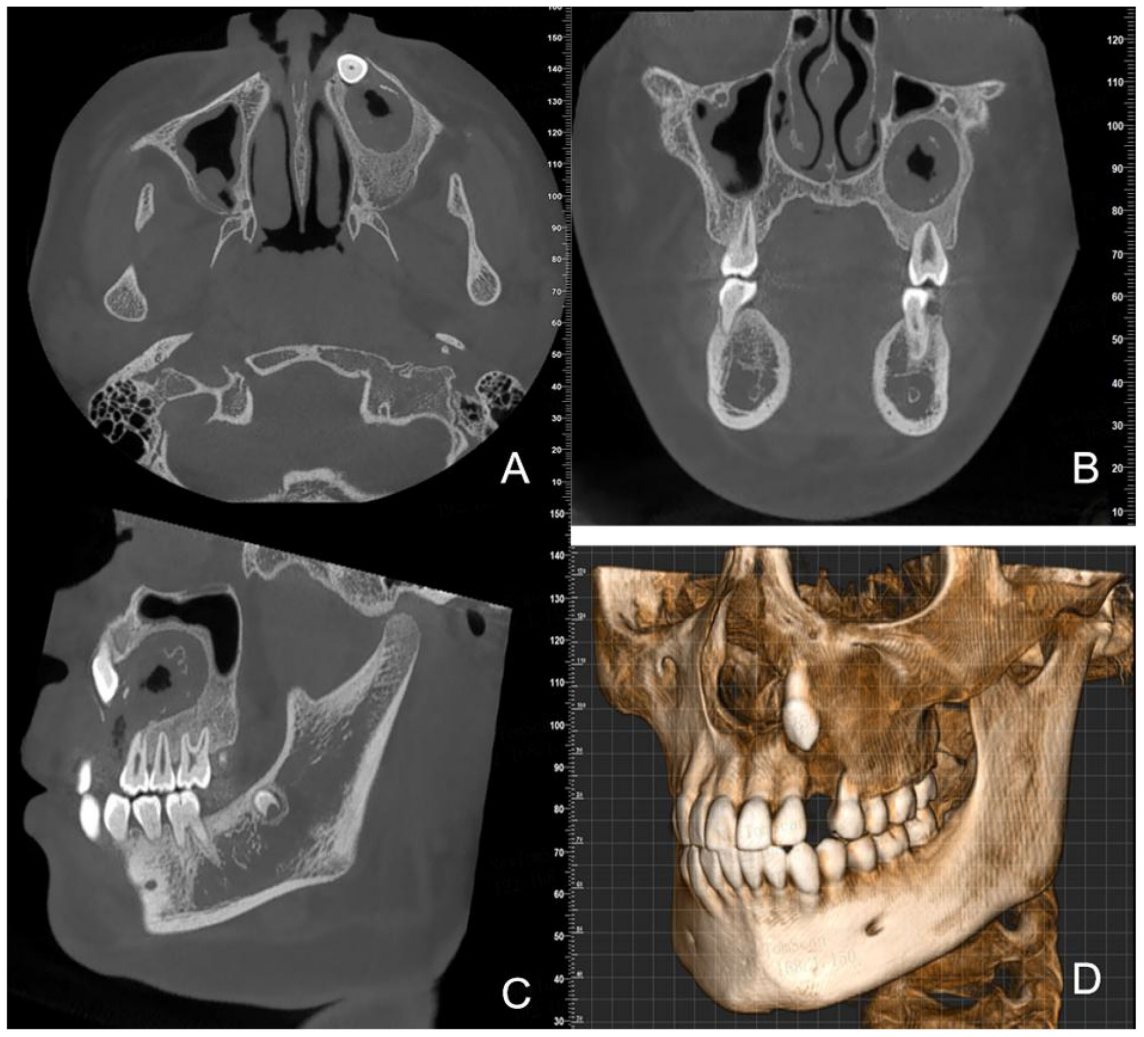
CBCT images one year after decompression surgery **(A)** (axial view) **(B)** (coronal view) **(C)** (sagittal view) **(D)** (3D reconstruction) show continued lesion reduction and ongoing bone repair.

One year after the decompression surgery, CBCT images showed that the lesion had shrunk to approximately 30mm×20mm×18mm. Ongoing bone repair was observed around the roots of teeth 24, 25, 26, and 27 (**Figure 4**). The family members clearly expressed that they were “satisfied with the current treatment progress and willing to continue cooperating with the follow-up surgery”.

The second surgical procedure was performed for “tumor resection + extraction of the impacted tooth 23”. Intraoperative observation revealed that the mass was cystic with thick walls and loss of content, and it was completely resected and sent for pathological examination.

**Figure 5:**
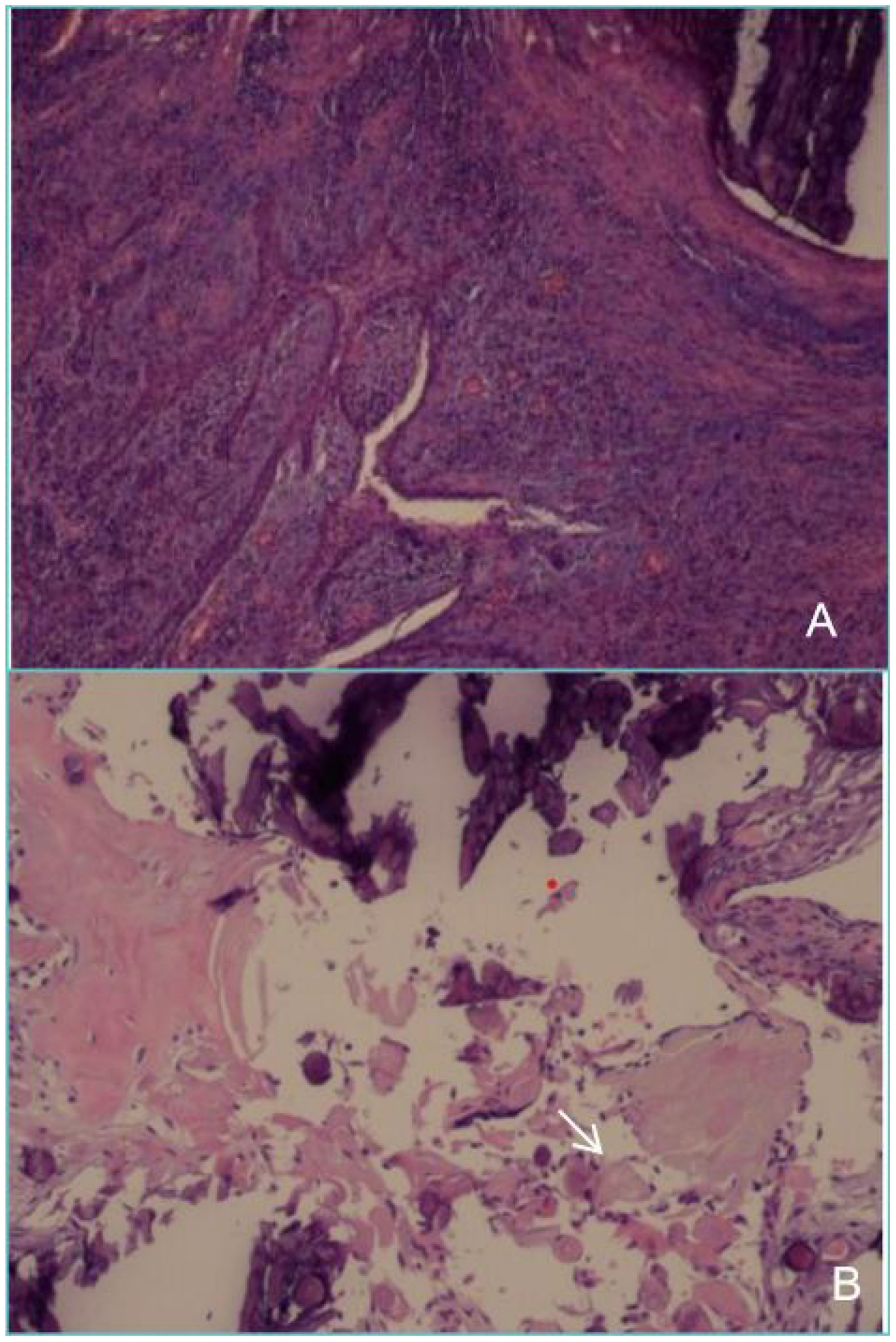
Postoperative pathology of the tumour resection **(A)** shows that the cyst wall is partly covered with squamous epithelium and partly with glaze, epithelium, with dentin hyperplasia, with extensive calcification. **(B)** shows shadow cells (white arrow) that are round or oval in shape, with distinct cell outlines. The cytoplasmic and nuclear staining has disappeared, leaving only empty shell-like shadow structures distributed around the calcified material.

**Figure 6:**
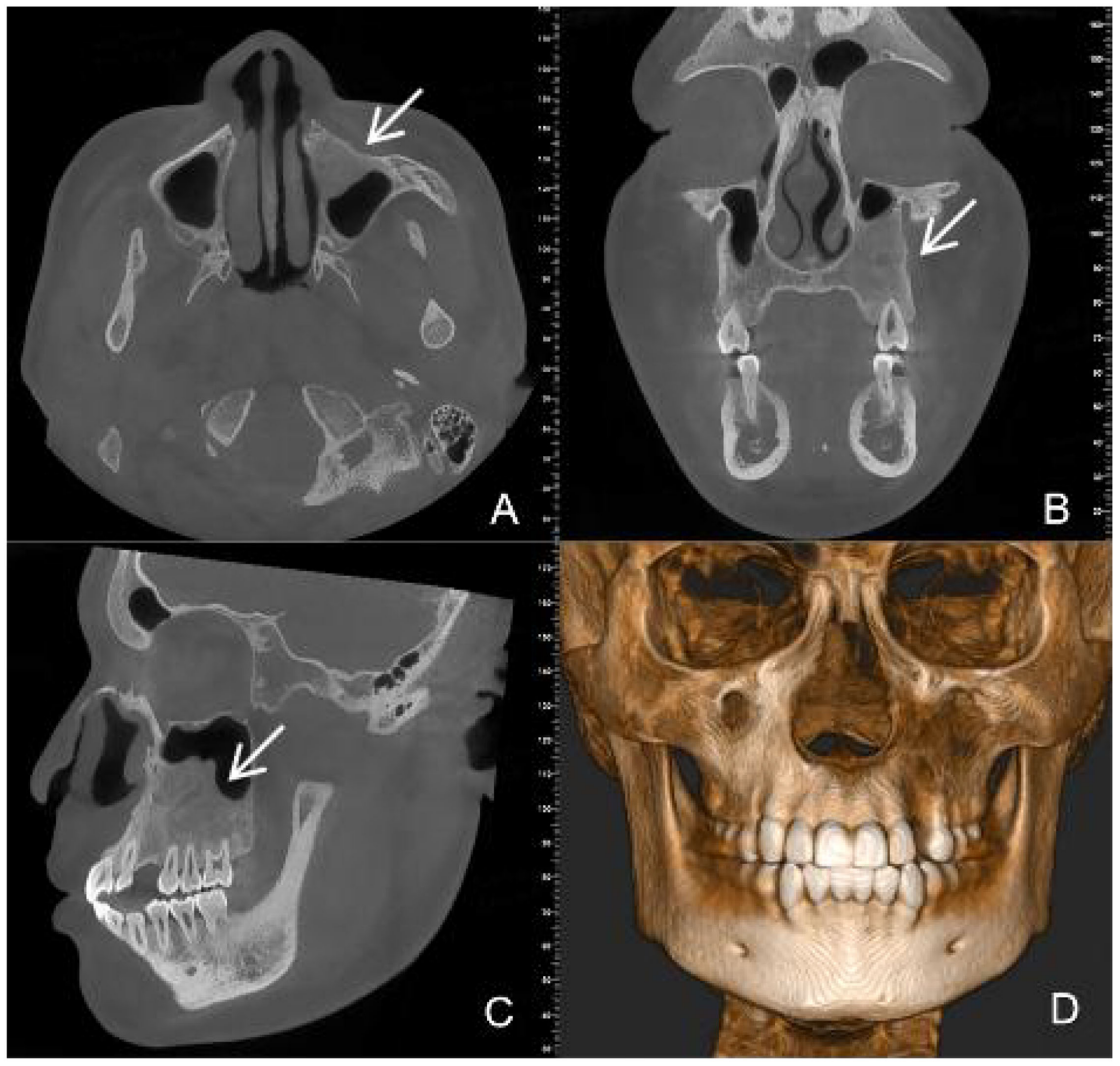
CBCT images at six months after tumour resection **(A)** (Axial view) **(B)** (Coronal view) **(C)** (Sagittal view) **(D)** (Three-dimensional reconstruction) show complete bone remodeling (white arrow).

Pathological examination revealed cystic wall-like structures within the fibrous tissue of the left maxillary bone. The cyst wall exhibited extensive infiltration by acute and chronic inflammatory cells, along with the development of inflammatory granulation tissue. The lining of the cyst wall was partially covered by squamous epithelium and partially by enamel epithelium, accompanied by dentinoid hyperplasia and widespread calcification. Additionally, foreign body giant cell infiltration and foreign body granuloma formation were observed in the cyst wall. Based on the histopathologic appearance, a diagnosis of intraosseous DGCT was given. (**Figure 5**). No recurrence or metastasis was observed during the six-month follow-up period. CBCT images at six months after tumour resection showed complete bone remodeling with no signs of recurrence; teeth 24, 25, 26, and 27 were preserved with vital pulp, indicating a good prognosis (**Figure 6**). One year postoperative follow-up showed no signs of recurrence. The patient expressed satisfaction with the treatment outcome.

## Discussion

DGCT was first described by Fejerslov and Krogh in 1972, initially referred to as odontogenic calcifying cystic odontogenic tumor (3). In 1981, Praetorius et al. suggested that the neoplastic odontogenic calcifying cystic odontogenic tumor should be named DGCT (4). In 2005, the WHO named the cystic variant as odontogenic calcifying cystic tumor; the solid variant as DGCT, and the malignant form as odontogenic ghost cell carcinoma (5). In 2017, the WHO renamed odontogenic calcifying cystic tumour as COC and classified it as a developmental cyst, and DGCT was classified as a benign mixed epithelial and mesenchymal odontogenic tumours (6). In 2022, the WHO continues to use this nomenclature (7).

DGCT is a rare benign odontogenic tumor. Its proportion in all odontogenic tumors is still less than 0.5%. DGCT is divided into intraosseous and extraosseous types, with the intraosseous type occurring in any part of the jawbone, 61.5% of which are found in the mandible (8). DGCT can occur in all age groups, with a relatively higher incidence rate in the elderly, and a male-to-female ratio of approximately 3:2, while it is extremely rare in children (2). According to literature reports, as of 2021, only one case of intraosseous DGCT has been reported in the 0-10 age group (8). In 2024, Yin YA reported another case of a 9-year-old boy suffering from this condition (9). Clinically, it can manifest as asymptomatic or facial asymmetry, swelling and pain in the cheek area, tooth displacement or loss, root resorption, etc.

In this case, imaging examinations played a significant role. CBCT images clearly showed the location, extent and nature of the lesion and monitored the progress of treatment, providing vital information for surgical planning. CBCT imaging revealed a mixed-density appearance, characterized by unilocular or multilocular radiolucent spaces in the jawbone, with irregularly sized high-density calcifications observed within them. The lesion often contains impacted teeth and can invade adjacent tissues, usually accompanied by root resorption. DGCT typically exhibits local aggressive growth but rarely metastasizes distantly.

The typical pathological features of DGCT include ameloblastic proliferation-like cells, ghost cells, and variable amounts of dentinoid matrix deposition, with more than 1%-2% of ghost cells and dentin formation being critical for the diagnosis of DGCT (10). Pathological findings in this case showed the presence of ghost cells, dentinoid material, and extensive calcification within the cyst wall, consistent with the diagnosis of calcifying odontogenic cyst. The initial biopsy was misdiagnosed as a calcifying odontogenic cyst, likely owing to insufficient tissue sampling post-decompression. Incomplete sampling obscures the lesion’s true morphology, raising the likelihood of diagnostic inaccuracies. The second biopsy, after complete excision of the mass, confirmed the diagnosis of DGCT.

Buchner analyzed 45 patients, among whom 21 mainly underwent conservative surgery such as excision or curettage, with 11 cases (73%) experiencing recurrence; 19 cases underwent radical surgery consisting of marginal or segmental resection, with 4 cases (33%) relapsing (11).At present, the main recommended treatment for DGCT is complete tumor resection with a surgical margin of ≥5mm (8). Rustemeyer adopted mandibular partial resection and temporary prosthesis repair for a 12-year-old child with DGCT involving the temporomandibular joint, and planned to carry out customized prosthesis repair after adulthood (12). This case adopted a phased treatment: considering the large range of the lesion, the initial operation performed decompression aimed at relieving symptoms and reducing tumor volume, while extracting deciduous teeth and supernumerary teeth. Post-operatively, the patient wore a cyst plug and had regular irrigation and dressing changes. Reexaminations after one and a half years showed a continuous and significant reduction of the lesion. Decompression is suitable for larger cystic lesions of the jawbone and achieved good clinical results in this case. However, Ye et al. (13) reported a case of DGCT in a 21-year-old adult, while Yin et al. (9) documented another case in a 9-year-old child. After decompressive fenestration of the tumor, the patient experienced increased postoperative swelling, and the lesion size showed no reduction. Therefore, in clinical practice, decompression should be performed only after proper evaluation of indications, followed by close monitoring. If the lesion continues to grow, immediate complete tumor resection or extended resection should be performed. In this case, the second operation performed tumor resection and extracted the impacted 23 tooth. The aim of this stage of surgery was to completely remove the lesion and reduce the risk of recurrence. As there is a possibility of malignant transformation or metastasis of the disease, close post-operative follow-up is required to monitor for recurrence. Follow-up of this case six months after tumour resection showed complete bone remodeling of the lesion without signs of recurrence. 24, 25, 26 and 27 were preserved, successfully avoiding autogenous bone repair after osteotomy. This effectively relieved the patient’s pain and significantly reduced the cost of subsequent implant repairs.

Pediatric patients are in a period of rapid development of the jawbones, teeth, and facial soft tissues. Complete tumor resection with a surgical margin of ≥5mm can lead to jawbone developmental deformities (such as jawbone hypoplasia, occlusal disorders), tooth loss, and even affect facial symmetry. This case suggests that preoperative decompression may represent an effective strategy for managing extensive DGCT tumors in select pediatric patients. Nevertheless, close clinical monitoring and long-term follow-up remain essential. We anticipate that these findings may offer valuable insights for oral and maxillofacial. Surgeons

## Data Availability

The original contributions presented in the study are included in the article/supplementary material. Further inquiries can be directed to the corresponding authors.
